# The nonsynaptic plasticity in Parkinson's disease: Insights from an animal model

**DOI:** 10.1016/j.clinsp.2023.100242

**Published:** 2023-07-20

**Authors:** Mônica P.C. Viegas, Luiz E.C. Santos, Mayra C. Aarão, Samyra G. Cecilio, Joana M. Medrado, Arthur C. Pires, Antônio M. Rodrigues, Carla A. Scorza, Marcelo A. Moret, Josef Finsterer, Fulvio A. Scorza, Antônio-Carlos G. Almeida

**Affiliations:** a Laboratory of Experimental and Computational Neuroscience, Department of Biosystems Engineering, Universidade Federal de São João del-Rei (UFSJ), São João del-Rei, MG, Brazil; b Neuroscience Discipline, Escola Paulista de Medicina da Universidade Federal de São Paulo (EPM/UNIFESP), São Paulo, SP, Brazil; c Centro de Neurociências e Saúde da Mulher “Professor Geraldo Rodrigues de Lima”, Escola Paulista de Medicina da Universidade Federal de São Paulo (EPM/UNIFESP), São Paulo, SP, Brazil; d SENAI ‒ Departamento Regional da Bahia, Centro Integrado de Manufatura e Tecnologia, Bahia, BA, Brazil; e Neurology & Neurophysiology Center Vienna, Vienna, Austria

**Keywords:** Parkinson's disease, Animal model, Non-synaptic mechanisms

## Abstract

**Background:**

The 6-OHDA nigro-striatal lesion model has already been related to disorders in the excitability and synchronicity of neural networks and variation in the expression of transmembrane proteins that control intra and extracellular ionic concentrations, such as cation-chloride cotransporters (NKCC1 and KCC2) and Na+/*K*+-ATPase and, also, to the glial proliferation after injury. All these non-synaptic mechanisms have already been related to neuronal injury and hyper-synchronism processes.

**Objective:**

The main objective of this study is to verify whether mechanisms not directly related to synaptic neurotransmission could be involved in the modulation of nigrostriatal pathways.

**Methods:**

Male Wistar rats, 3 months old, were submitted to a unilateral injection of 24 µg of 6-OHDA, in the striatum (*n* = 8). The animals in the Control group (*n* = 8) were submitted to the same protocol, with the replacement of 6-OHDA by 0.9% saline. The analysis by optical densitometry was performed to quantify the immunoreactivity intensity of GFAP, NKCC1, KCC2, Na+/*K*+-ATPase, TH and Cx36.

**Results:**

The 6-OHDA induced lesions in the striatum, were not followed by changes in the expression cation-chloride cotransporters and Na+/*K*+-ATPase, but with astrocytic reactivity in the lesioned and adjacent regions of the nigrostriatal. Moreover, the dopaminergic degeneration caused by 6-OHDA is followed by changes in the expression of connexin-36.

**Conclusions:**

The use of the GJ blockers directly along the nigrostriatal pathways to control PD motor symptoms is conjectured. Electrophysiology of the striatum and the substantia nigra, to verify changes in neuronal synchronism, comparing brain slices of control animals and experimental models of PD, is needed.

## Highlights

• 6-OHDA lesioned striatum without changes in CCC cotransporters and Na+/*K*+-atpase.
• However, was observed astrocytic reactivity.
• Dopaminergic degeneration was followed by changes in connexin-36.

## Introduction

Parkinson's Disease (PD) is the second most common neurodegenerative disorder of aging after Alzheimer's disease and one of the most common movement disorders.^1^ In 2016, 6.1 million individuals had PD globally and caused 211,296 deaths.^2^ There are more than 10 million people in the world living with PD and in the last two decades, a significant increase in the death rate in individuals with this disease has been observed.^3^ In addition, PD is estimated to increase by more than 50% by 2030, and the only proven risk factor for idiopathic PD is advancing age.^1^^,^^4^ PD is clinically characterized by tremor, rigidity, bradykinesia/akinesia, and postural instability, but the clinical picture includes other motor and non-motor symptoms.^5^ Movement abnormalities in PD are related to the loss of Dopaminergic neurons (DA) of the *Substantia Nigra pars compacta* (SNpc) and widespread intracellular aggregates of α-synuclein.^6^ PD's available medical therapies only manage disease symptoms and predominantly focus on the dopaminergic pathway, but do not halt the progression of neurodegeneration and the evolution of the disease.^6^ Although the authors are witnessing an illuminating period in PD research,^1^ the exact molecular and cellular basis of PD is still unclear, particularly synaptic and non-synaptic mechanisms. While synaptic functions are responsible for definite, accurate tasks that require high speed and precision; non-synaptic functions are related to the modulation and tuning of these processes.^7^ Several studies have focused their efforts on elucidating synaptic dopaminergic mechanisms in the pathophysiology of PD. Pathological beta-band oscillatory activity in the basal ganglia has been extensively described in PD subjects and suggested as a mechanism that subserves motor dysfunction.^8^ The nigro-striatal lesion model, which makes use of the 6*-*Hydroxydopamine (6*-*OHDA) neurotoxin to reproduce parkinsonism, has already been related to disorders in the excitability and synchronicity of neural networks in beta and gamma frequencies.^9^ Dynamic changes in the basal ganglia network are critical in PD pathophysiology, including changes in firing patterns, particularly in oscillatory activity, in addition to variation in average firing rates.^10^ Interestingly, studies in animal models of PD suggest that one of the consequences of the loss of nigrostriatal dopaminergic inputs is the increased oscillatory triggering and synchronization in the Subthalamic Nuclei (STN).^10^ In these lines, the injuries caused by the 6-OHDA neurotoxin could induce changes in the synchronizing mechanisms of motor control, with an imbalance between the inhibitory and excitatory activities of neurons and interneurons activated in groups, through the variation in the expression of transmembrane proteins and control of intra and extracellular ionic concentrations, such as cation-chloride cotransporters (NKCC1 and KCC2) and Na^+^/*K*^+^-ATPase and, also, through the glial proliferation after injury. All these non-synaptic mechanisms have already been related to neuronal injury and hyper-synchronism processes, as described in diseases such as epilepsy, traumatic brain and spinal cord injuries, and PD.^11^, ^12^, ^13^, ^14^ Therefore, knowing the alterations in the expression of proteins linked to these non-synaptic activities could assist in understanding the pathological changes associated with dopaminergic nigrostriatal depletion in experimental PD and could lead to the prospect of new pharmacological targets for treatment and care in PD.

The main objective of this study is to verify whether mechanisms not directly related to synaptic neurotransmission could be involved in the modulation of nigrostriatal pathways. The hypothesis raised is that changes in the expressions of these non-synaptic mechanisms could unbalance the functions mediated by GABA on nigrostriatal circuits, leading to the initiation and execution of the neurodegenerative process and symptoms of PD.

## Materials and methods

### Ethical standards

All procedures applied in this investigation were approved (protocol number 012/2018) by the Institutional Animal Care and Use Ethical Committee, Federal University of São João del-Rei, Brazil. All institutional guidelines ruling research involving animals were followed. These guidelines are in accordance with ARRIVE guidelines. The animals were kept on a 12h light/dark cycle with controlled temperature (21 ± 2 °C) and humidity (50%‒55%). Water and food were provided ad libitum.

### Animals

Male Wistar rats (*Rattus norvegicus*), 3 months old, were randomly divided into two groups, according to the treatment they were subjected to. The animals of the DP24 group (*n* = 8), were submitted to a unilateral injection of 24 µg of 6-OHDA, in the striatum. The animals in the Control group (*n* = 8) were submitted to unilateral injection under the same conditions, with the replacement of 6-OHDA by 0.9% saline.

### Surgical procedures

All animals were submitted to stereotaxic surgery. For surgery, the animals were anesthetized with a mixture of ketamine hydrochloride (100 mg/kg, via ip, Dopalen®), xylazine hydrochloride (7.5 mg/kg, via ip, Anasedan®), and fentanyl citrate (2 mg/kg, via ip, Fentanyl®) and submitted to trichotomy of the dorsal region of the head and positioned in the stereotaxic apparatus (insight model EEF 331). Then, the animals were subjected to a solution of iodized alcohol for asepsis, and a small amount of lidocaine and norepinephrine (2%) was applied subcutaneously in the region of the scalp to be opened in order to avoid hemorrhage. Subsequently, an anteroposterior incision was made to expose the skull and locate the bregmatic and lambda suture. Cotton soaked in hydrogen peroxide was used to remove the remaining layer of subcutaneous tissue and allow better visualization of the sutures. After demarcating the stereotaxic coordinates, the skullcap was drilled with a dental drill. Although the structure of 6-OHDA is like that of dopamine, the presence of an additional hydroxyl group makes it toxic to dopaminergic neurons. This compound does not cross the blood-brain barrier, which requires its direct injection into the Substantia Nigra par compacta (SNpc), Medial Forebrain Bundle (MFB), or striatum.^15^ For this study, the injection of 6-OHDA to induce PD was performed in two positions, in the striatum, following the coordinates: AP: Bregma LL: −2.7 mm; DV: −4.5 mm and AP: 0.5 mm LL: −3.2 mm; DV: −4.5 mm.^16^ All injection coordinates were calculated according to the stereotactic atlas of the rat brain.^17^ To infuse the drug, a gingival needle attached to a polyethylene cannula was used, which allows the connection between the Hamilton syringe and the needle. The syringe was filled with the 6-OHDA solution. 6-OHDA must remain very well maintained and completely free of any moisture. On the same day of the infusion, the drug was dissolved in ascorbic acid 0.3% and subsequently injected in a volume of 0.5 µL, through a continuous infusion pump, for 2 minutes, then waiting for a 5-minute period for the diffusion of the substance, in each of the coordinates. Immediately after the skin of the skull was sutured and the animal was placed under observation until it was fully awake. After surgery, the animals received a prophylactic intramuscular injection of antibiotic (enrofloxacino ‒ 93,106–60–6). After 15 days after PD induction, the animals' brains were evaluated by immuno-histochemical/immunofluorescence analysis, with appropriating staining for tyrosine hydrolysis, cation-chloride co-transporters, parvalbumin, Na^+^/*K*^+^-ATPase, and glial cells. The choice of this time period was in line with reported findings,^18^ that demonstrated that intra-striatal injections of 6-OHDA cause a biphasic course of neurodegeneration, with a rapid phase of dopaminergic cell death occurring within 1 week in the rat. This phase is followed by 4‒6 weeks of additional mild loss of nigral DA neurons.

### Histological analysis by immunofluorescence

To determine Tyrosine Hydrolase (TH) immunoreactivity, glial reactivity, and variation in expression of cotransporters (NKCC1 and KCC2), Na^+^/*K*^+^-ATPase and connexins 36, animals in groups 24DP (*n* = 8) and Control (*n* = 8) were euthanized by anesthetic overdose (ketamine-xylazine 100 mg/kg – 5 mg/kg, respectively) followed by trans-cardiac perfusion with phosphate buffer solution (0.1 M Phosphate Buffered Saline [PBS], pH = 7.4) followed by paraformaldehyde 2% in PBS 0.1 M. The brain was removed and remained immersed in paraformaldehyde 2% overnight and after post-fixation, were bathed in PBS and stored at 4 °C until sectioning in 40 µm slices by means of a vibratome (Leica Microsystems, Germany). The coronal slices of the regions close to the injection of 6-OHDA, rostral-caudal direction, from 2.0 to −0.4 mm, with the bregma suture as a reference, were bathed for 4 h, at room temperature, in blocking solution nonspecific sites (10% bovine serum albumin in 0.1 M PBS and 0.3% Triton X-100). Then, the sections were incubated for 48 h, at 4 °C, in solution (2% bovine albumin serum in 0.1 M PBS and 0.1% Triton X-100) containing the primary anti-GFAP antibodies (rabbits polyclonal antibody for astrocyte; 1:1000; Abcam) or anti-NKCC1 (mice monoclonal antibody for NKCC1 cotransporter; 1:50; T4, DSHB) or anti-TH (mice monoclonal antibody for tyrosine hydrolase; 1:5000; Incstar) or anti-α1- Na^+^/*K*^+^-ATPase (mice monoclonal antibody for alpha 1 subunit; 1:50; a6f, DSHB) and anti-KCC2 (rabbit polyclonal antibody for KCC2 cotransporter; 1:1000; Abcam) or anti-Cx36 (rabbit polyclonal antibody for connexin-36; 1:100; Abcam). After the process with the primary antibodies, the sections were incubated for 2 h, at room temperature, in solution with the secondary anti-rabbit IgG antibodies (goat polyclonal antibody, conjugated with the fluorophore DyLight® 488; 1:250, Abcam) and anti-mouse IgG (goat polyclonal antibody, conjugated with the fluorophore DyLight® 594, 1:250; Abcam). To test the specificity of the antibodies and possible interferences of tissue autofluorescence, some slices were subjected to incubations where the primary antibodies were omitted. The sections were mounted on glass slides coated with gelatin and covered with coverslips deposited using glycerol. The photomicrographs obtained from 2–4 sections per animal cut separated by about 200 µm, were obtained using a confocal microscope (Zeiss LSM 710) equipped with a primary beam splitter of 488/543, using an argon laser of 488 nm for the secondary antibody conjugated to the fluorophore Dylight® 488 and a helium-neon laser of 543 nm for the secondary antibody conjugated to the fluorophore Dylight 594®. The images were captured with a 10 ×  lens for a complete reconstruction of the brain slice, creating an image of approximately 13,000 × 8000 pixels (104 megapixels). From the high-resolution photomicrograph, the regions most affected by the striatal lesion promoted by 6-OHDA were identified, using regions with low or no immunoreactivity to TH as a reference. These same regions were observed on the slices containing the other markings, guaranteeing the capture of the images in the same regions where the loss of dopaminergic fibers occurred. After checking the regions of injury in the animals, the photomicrographs were captured with a 63 × glycerol immersion objective (Plan-Apochromat 63 × /1.40 Oil Dic M27) for analysis of optical densitometry. The pinhole was configured for both magnifications in one airy unit.

### Optical densitometry

The analysis by Optical Densitometry (OD), as described in,^19^ was performed to quantify the immunoreactivity intensity of GFAP, NKCC1, KCC2, Na^+^/*K*^+^-ATPase, TH, and Cx36. Due to the dispersed characteristic of PV immunostaining, it was not possible to evaluate the regions with the greatest cell proliferation by optical densitometry, however, a qualitative analysis was carried out in all groups. The photomicrographs obtained with the 63 ×  objective (225 µm^2^; 2‒5 per region of interest) were sampled in both hemispheres (ipsilateral and contralateral to the lesion), for each staining. The confocal photomicrographs were processed in RGB and compressed to grayscale (RGB mean band), to obtain the corresponding histograms. To improve the contrast, a histogram equalization technique was incorporated into the analysis process.^20^ In order to avoid the interference of the autofluorescence of lipofuscin deposited on neurons, its spectrum was digitally subtracted from the samples of each photomicrograph collected. The resulting images provided an intensification of the pixels corresponding to the staining, which resulted in more reliable segmentations. A grayscale interval (between 0 and 65 in grayscale) was defined as a threshold for considering the immunoreactive area of the tissue. The significant pixels were later converted into a binary matrix (black and white) and quantified by the sum of black pixels per area. Quantification was performed using a computer system developed in MATLAB, with photomicrographs showing high resolution and the same optical zoom. The data were plotted as a percentage of equivalent immunoreactivity for each photomicrograph.

### Statistical analysis

Quantitative measurements, carried out by means of optical densitometry, of immunoreactivities to GFAP, NKCC1, KCC2, α1- Na^+^/*K*^+^-ATPase and Cx36, of striatal regions referring to the area with loss of immunoreactivity to TH, were compared to the control group and to the contralateral side of the animals in the DP24 group. The Shapiro-Wilk test was applied to the OD values of the areas of loss of immunoreactivity to TH (lesion area) and areas external to the lesion to analyze the sample distribution. Given the normal distribution of the data (*p* < 0.05), a one-way analysis of variance (one-way ANOVA) was applied, followed by Tukey's multiple comparison tests to assess OD. For the analysis of GFAP expression in the regions outside the lesion, in the striatum, *t*-Student test for unpaired samples (unp-TT) was applied. All data were presented as means ± Standard Error of the Mean (SEM). For all cases, a significance level of 5% was adopted.

## Results

### The injection of 6-OHDA induced lesions in the striatum

**Fig. 1:**
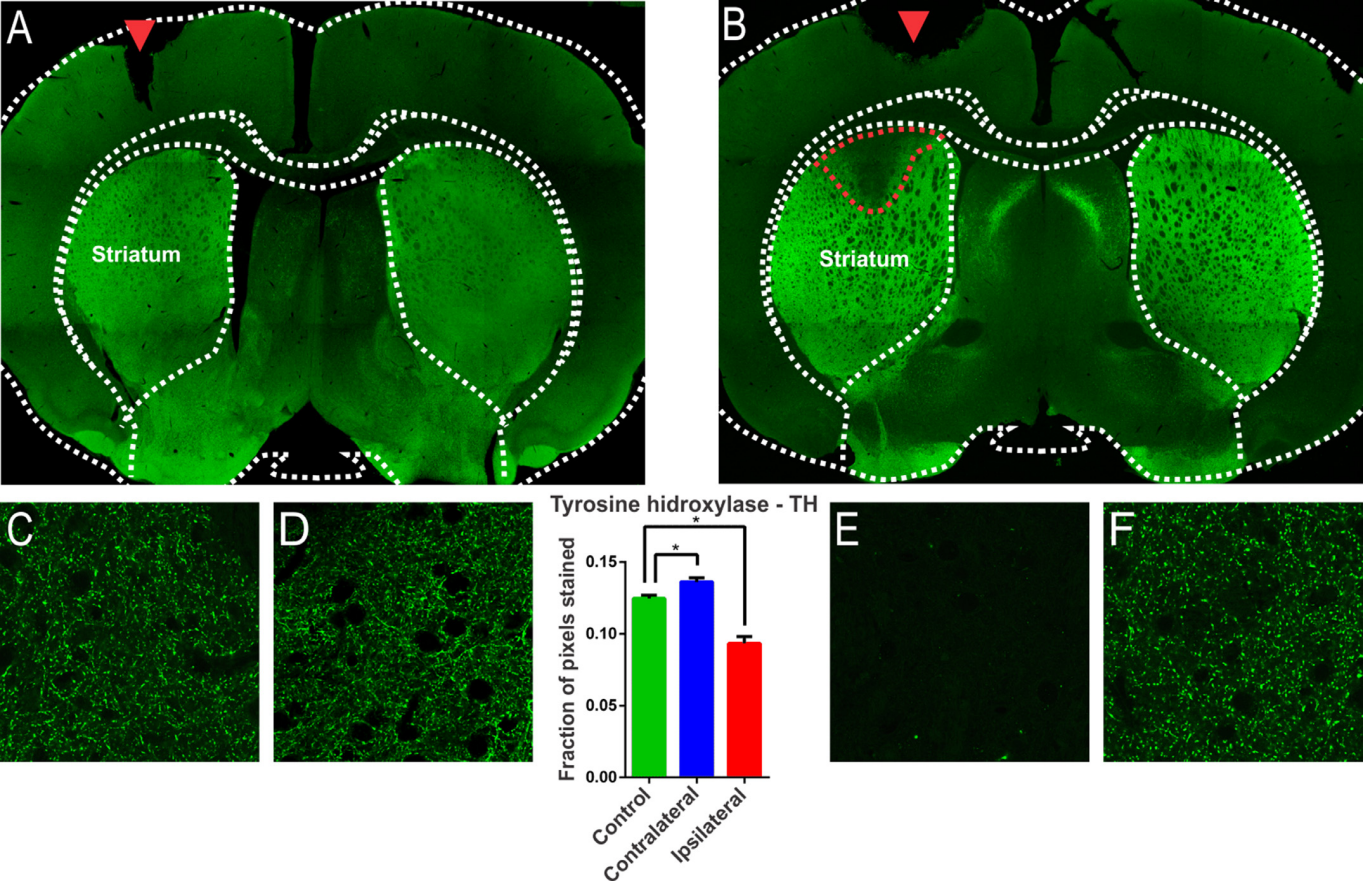
Confocal photomicrograph of coronal section of the striatum region of Wistar rat after injection with saline (A) and 6-OHDA (B). Observe, in B, in a region delimited by a red dotted line, the massive reduction in tyrosine hydroxylase (TH) immunoreactivity in the upper lateral portions. In (A), only the lesion on the injection needle is observed (red arrow). In (E), the region of injury by 6-OHDA is observed in greater magnification, where there is a decrease in the number of dopaminergic fibers, evidenced by the reduction of immunoreactivity. Images (C), (D) and (F) show the presence of dopaminergic neurons, similarly, on the contralateral side and in the animals of the control group. The single-factor analysis of variance for the DO data of the photomicrographs showed significant differences between the groups evaluated, confirming the qualitative observation of the photomicrographs. * Indicates *p* < 0.05.

A characteristic of the 6-OHDA injection model is the extensive oxidative damage caused in the nigrostriatal pathway.^21^ The extent of the lesion can be verified through the location of the enzyme Tyrosine Hydrolase (TH), evidenced in dopaminergic terminals.^21^ Therefore, brain sections of the animals of the experimental groups were subjected to immunostaining for TH according to the 6-OHDA injection protocol. 6-OHDA treated animals showed an area with massive reduction (almost to the background) in immunoreactivity to TH in the upper lateral portions of the striatum (Fig. 1). The animals in the control group, which were submitted to the injection of saline in substitution of 6-OHDA, presented only the injury due to the injection needle (Fig. 1, inlet lesion highlighted by the blue rectangle). The single-factor analysis of variance for the OD data from the photomicrographs showed a significant difference between the groups evaluated (Lesion area: 0.093 ± 0.004 pixel fraction per area; Contralateral area: 0.136 ± 0.003 pixel fraction per area; Control: 0.125 ± 0.002 fractions of pixels per area; F(2.21) = 40.98; *p* < 0.0001, ANOVA). These results demonstrated that the protocol was successfully performed. Through the delimitation of the lesion area, evidenced by the immunoreactivity to TH, slices were examined adjacent to the injection point to verify the immunoreactivity to structures related to non-synaptic mechanisms.

### Expression of striatal cation-chloride cotransporters and Na^+^/*K*^+^-ATPase are not affected by 6-OHDA neurotoxin

**Fig. 2:**
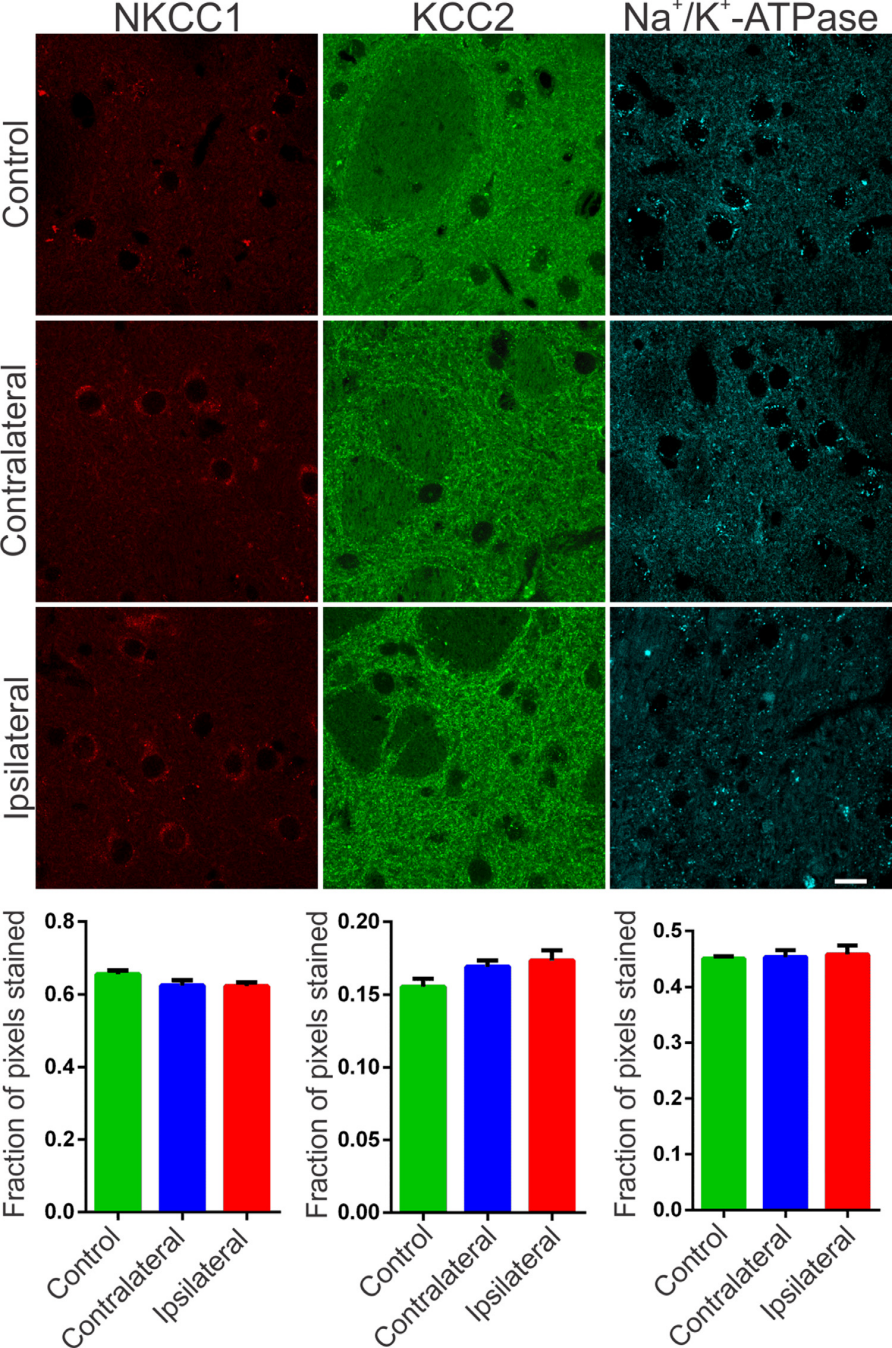
Representative photomicrographs of immunoreactivity to NKCC1 (red), KCC2 (green) and Na^+^/*K*^+^-ATPase (cyan blue) of Wistar striatal region after 6-OHDA injection – ipsilateral (lesional region) and contralateral – and sections after saline injection (control animals). For NKCC1 immunostaining, sparce immunoreactivity is observed and also around large cell bodies, characteristic of spiny neurons of the mature striatal nucleus. For KCC2, a difuse staining is observed and, mainly, with a puncta pattern throughout the striatum. Immunofluorescent puncta is also typical of the α1 subunit of the Na^+^/*K*^+^ -ATPase enzyme staining. No significant variation was observed between the regions injured with 6-OHDA and other regions of the striatum, as can be seen in the representative images and confirmed by optical densitometry quantification and comparative analysis. White bar indicates 10 µm.

**Fig. 3:**
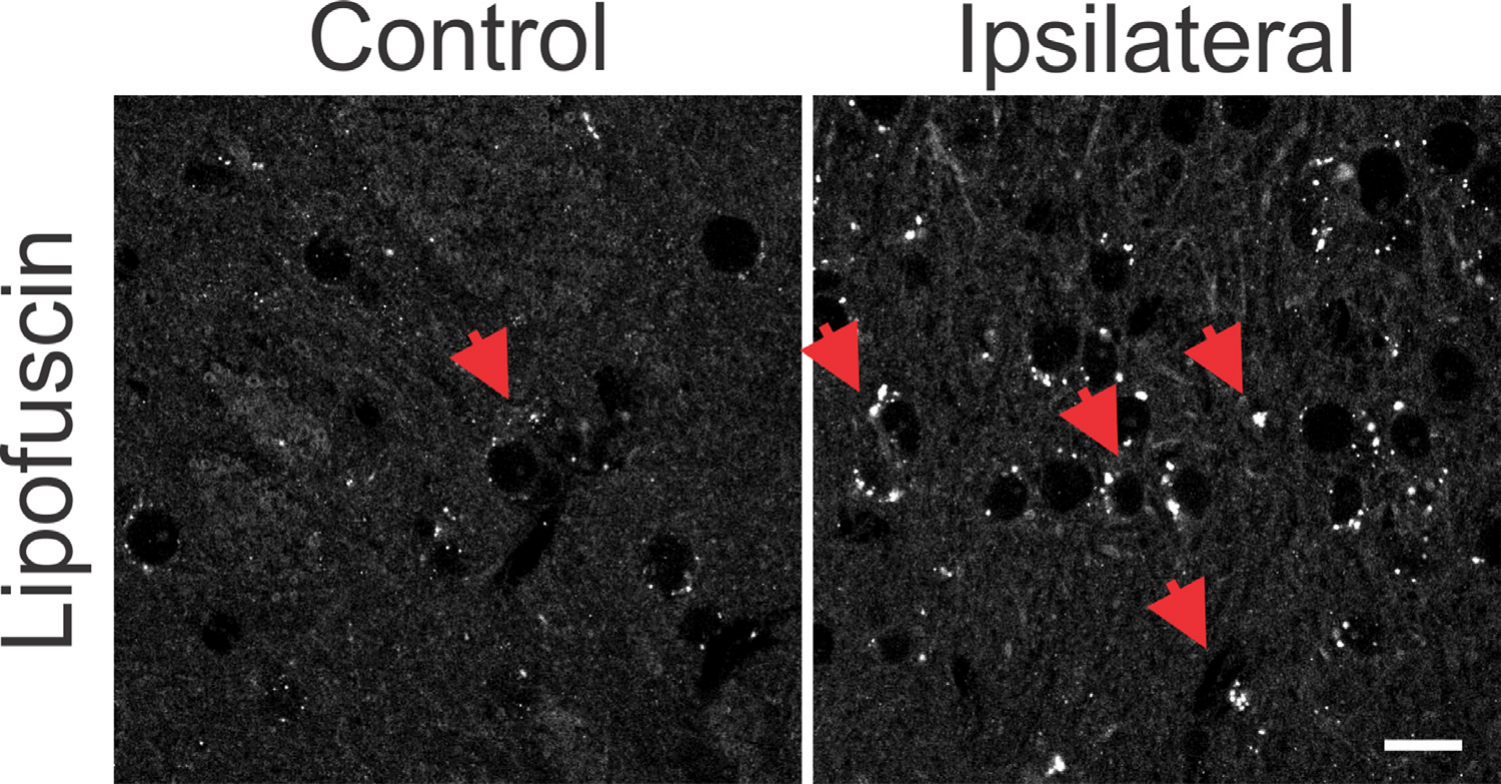
Photomicrographs with omission of primary antibodies in striatal regions after 6-OHDA injury and in control animals. Observe the punctuated aspect of cytoplasmic depositions similar to the autofluorescent lipofuscin pigment (red arrows). The lesional region shows more intense punctuated areas when compared to the same region without the 6-OHDA lesion, in the control sections. There is also a greater number of cells with a small cell body, similar to glial cells in the lesional region. White bar indicates 10 µm.

The synchronization of neuronal populations in the striatum is regulated by the fine excitatory and inhibitory control mediated by the joint activity of the populations of GABAergic spiny neurons and interneurons. This is the basic mechanism responsible for preventing normal synchronization from becoming unregulated, as demonstrated in PD.^9^^,^^21^ The chronic dopamine depletion, caused by the nigrostriatal lesion caused by 6-OHDA, can affect the activity of these cells, generating giant depolarizing potentials.^20^ The efficiency of GABAergic inhibition is dependent on the intracellular level of chloride concentration.^20^^,^^22^ When elevated, it can take the effect of GABA from hyperpolarizing to depolarizing. The intracellular chloride concentration is primarily regulated by the chloride cotransporters KCC2 and NKCC1.^22^ NKCC1 is responsible for carrying chloride to the intracellular space and when blocked with bumetanide in PD patients results in attenuation of motor symptoms.^14^ Therefore, it is important to verify if the nigrostriatal lesion by 6-OHDA could be associated with the altered expression of the co-transporters NKCC1 and KCC2 in the injured regions. Immunoreactivity to NKCC1 in the lesioned striatum as well as in the contralateral one was as diffuse as in the control group. There is slightly more intense staining observed in peri‑somatic or perinuclear regions (Fig. 2, left), but with no relevant differences when compared to the control group. Likewise, the analysis by optical densitometry did not show significant differences in immunoreactivity to NKCC1 between the lesion and contralateral areas of the DP24 group itself, as well as when compared with the corresponding areas of the control group (lesion area: 0.622 ± 0.011 fraction of pixels per area; contralateral area: 0.624 ± 0.015 pixel fraction per area; control: 0.656 ± 0.011 pixel fraction per area; F(2.27) = 2.154; *p* = 0.1356, ANOVA). The immunoreactive staining to KCC2 was intense in all groups analyzed. Throughout the region of the striatal area, intense peri‑somatic and dendritic process staining were observed. Photomicrographs of KCC2 immunoreactivity in the striatum of both groups are shown in Fig. 2 (middle). Comparing the optical densitometries, there was no significant difference in the expression of KCC2 in the animals subjected to the 6-OHDA lesion in comparison to their contralateral region or to the Control group (Area of the lesion: 0.1734 ± 0.007 fraction of pixels per area; Contralateral area: 0.169 ± 0.004 pixel fraction per area; control: 0.155±0.005 pixel fraction per area; F(2.31) = 2.487; *p* = 0.0996, ANOVA). The energy for ionic transport mediated by co-transporters is derived from ionic gradients generated by the action of Na^+^/*K*^+^-ATPase. This is why co-transporters indirectly use ATP hydrolysis energy.^20^ Therefore, it is necessary to investigate changes in the expression of Na^+^/*K*^+^-ATPase to interpret the effects on the action of co-transporters, even when their expressions are not altered. Therefore, the immunoreactivity to the α1 subunit of Na^+^/*K*^+^-ATPase was evaluated in the striatum of the slices of the studied groups. Immunoreactive puncta and moderate non‑peri-somatic staining were observed, and this characteristic was present in all groups analyzed. As shown in Fig. 2 (right), there was no statistically significant difference in the expression of this protein between the groups evaluated by univariate analysis of the optical densitometry (area of the lesion: 0.458 ± 0.015 pixel fraction per area; contralateral area: 0.4536 ± 0.012 pixel fraction per area; control: 0.451 ± 0.004 pixel fraction per area; F(2.33) = 0.1017; *p* = 0.9036, ANOVA). The specificity of immunoreactivity to co-transporters and α1- Na^+^/*K*^+^-ATPase was evaluated using negative controls, with the omission of primary antibodies from animal slices in both groups. No staining was observed like those seen on the immunostained slices. However, it was observed the presence of autofluorescent granules, intra and extracellular, characterized by small globular aggregates and with a wide range of excitation, emitting in long spectrum red. These aggregates are similar to lipofuscin, a pigment common in mature neurons, formed from organelles debris, remnants of cellular metabolism, or oxidative stress, accumulating in the cytoplasm.^23^ Small spots like lipofuscin, distributed around the nucleus, in the cytoplasm, were observed in control slices. However, in slices of the DP24 group, the observed aggregates were much larger, occupying a large cytoplasmic area. In addition, cells with smaller nuclei (possibly glial) had bulky autofluorescent aggregates close to the nucleus (Fig. 3).

### The 6-OHDA administration induces astrocytic reactivity in the lesioned regions and in the regions adjacent to the nigrostriatal lesion

**Fig. 4:**
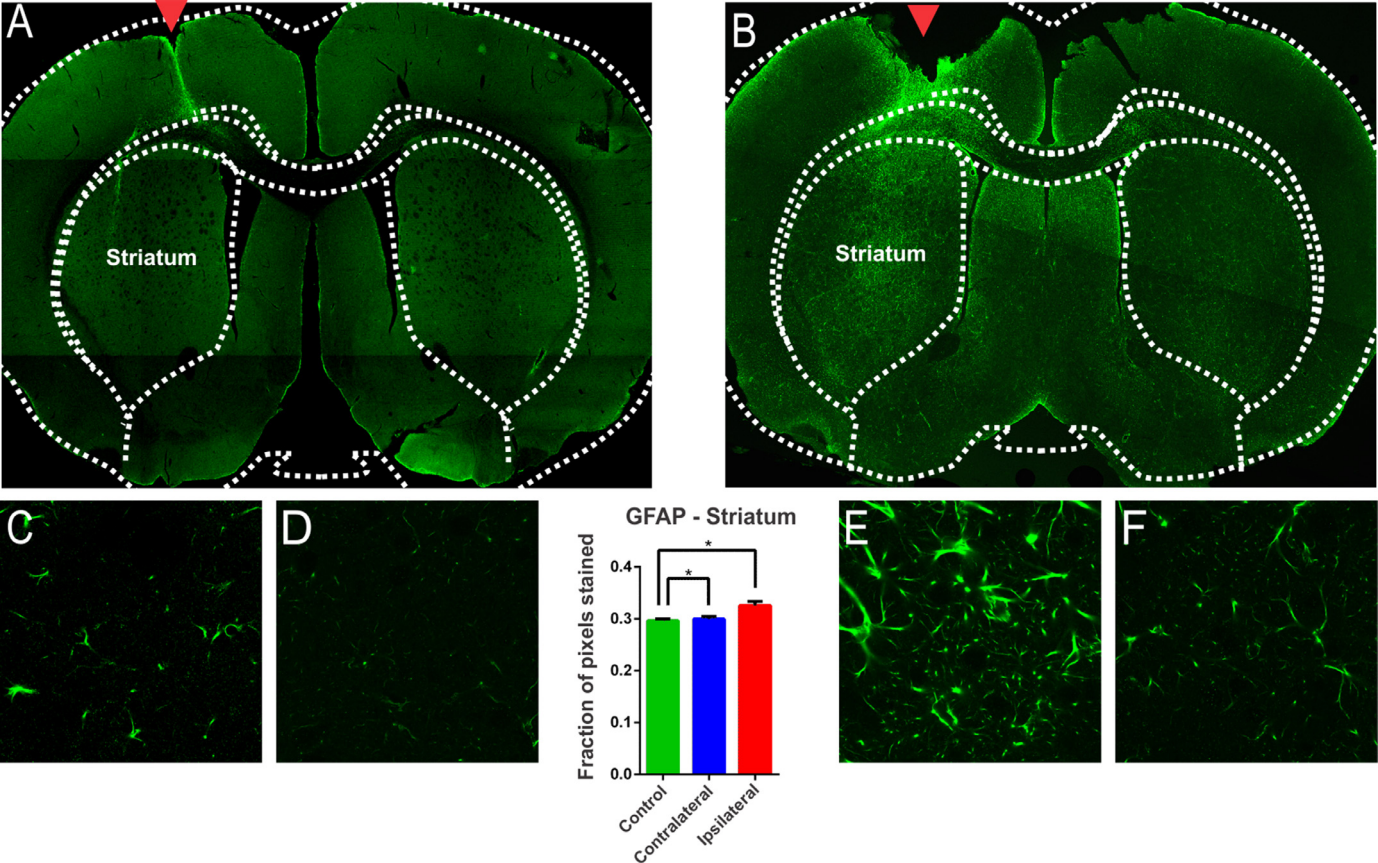
Evaluation of astroglial reactivity in the striatal injured region investigated with immureactivity to GFAP in the groups submitted to the injection of 6-OHDA (B) and in the Control group (A). The GFAP staining is clear in (B), with the presence of reactive/hypertrophic astrocytes with large cell bodies and thick cytoskeletal processes, which can be seing in higher magnification (E). The monofactor analysis of variance of the optical densitometry data showed a significant increase in the intensity of GFAP staining in the lesional region, confirming the observation verified in E, in relation to the contralateral region (F) and the control group (C and D). The quantitave analysis shows that the glial reactivation process occurred specifically due to the 6-OHDA, and not due to the lesion promoted by the injection, as can be seen in Figures (C) and (D) and the statistical data. GFAP immunoreactivity is also observed in reactive astrocytes in regions adjacent to the lesion observed by TH immunoreactivity (Fig. 1), which demonstrates a spread of astrocytic reactivity processes. * Indicates *p* < 0.05.

The glial reaction is commonly seen after injury to the Central Nervous System (CNS).^24^ Hyperplasia and hypertrophy of microglia and astrocytes are described after brain injury, depending on the temporal phase in which the samples are analyzed.^24^ From the morphological point of view, astrocytic reactivity consists of an increase in the glial cells’ body and an increase in the number and size of the astroglial processes.^24^ In addition, this reactivity is accompanied by a positive regulation of Glial Fibrillar Acid Protein Synthesis (GFAP). There are many publications on the degeneration of the nigrostriatal system after a nigral stereotactic injection of 6-OHDA. However, some studies do not clearly demonstrate whether the glial involvement is due to the injection procedure itself or the 6-OHDA injury protocol. In this study, intense GFAP staining was observed in animals submitted to 6-OHDA injection showing reactive/hypertrophic astrocytes with large cell bodies and thick cytoskeletal processes (Fig. 4). The optical densitometry showed a significant increase in the intensity of GFAP staining in the ipsilateral region of the DP24 group when compared to the contralateral region and to the control group (lesion area: 0.326 ± 0.007 pixel fraction per area; contralateral area: 0.299 ± 0.005 pixel fraction per area; control: 0.296 ± 0.003 pixel fraction per area; F(2.27) = 8.145; *p* = 0.0017, ANOVA). These results demonstrate that the glial reactivation process occurred specifically due to the injection of 6-OHDA, and not due to the injection lesion, according to the Tukey multiple comparison test (Fig. 4). The regions with intense reactive astrocyte staining were not limited to the region of the nigrostriatal lesion. GFAP staining was also observed in reactive astrocytes in regions adjacent to the lesion characterized by TH immunoreactivity, which demonstrates a spread of astrocytic reactivity processes, with a high incidence of lipofuscin, throughout the striatum (Fig. 4). To verify whether these areas were also significantly different from ipsilateral nigrostriatal lesion regions, slices of the animals of the DP24 group were analyzed by optical densitometry performed in the regions outside of the lesioned areas, on its contralateral sides. This analysis, performed by means of the *t*-Student test with unpaired samples (unp-TT), showed a significant difference even between regions adjacent to the lesion, confirming the qualitative findings (regions adjacent to the lesion area: 0.317 ± 0.004; contralateral regions: 0.299 ± 0.004; *t* = 3.001; df = 90; *p* = 0.0035, unp-TT).

### The dopaminergic degeneration caused by 6-OHDA is associated with changes in the expression of connexin-36

**Fig. 5:**
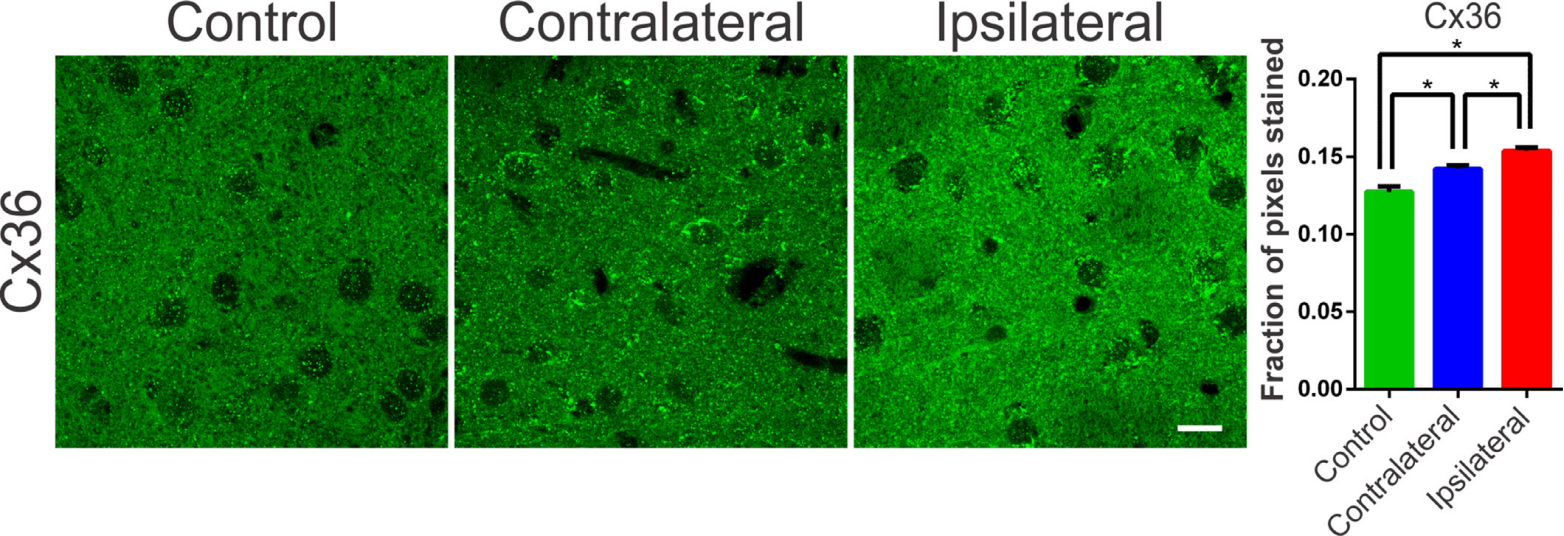
Typical photomicrographs of Connexin 36 (Cx36) immureactivity in the striatum regions of the groups submitted to the 6-OHDA injection (Injured and contralateral area) and in the Control group. Note the Cx36 stainning with a immunoreactive puncta throughout the region of dendritic fibers and around the cell body of striatal neurons. The lesional region showed more intense immunoreactivity along its entire length, with zones of greater intensity around the cell body of the cells observed. The contralateral region also showed an increase in the staining intensity, mainly perisomatic, when compared to the control sections. The monofactorial analysis of variance of the optical densitometry data confirmed this significant increase in the intensity of Cx36 staining in the lesional region and in the contralateral striatal region, in relation to the control group. White bar indicates 10 µm. * Indicates *p* < 0.05.

Communication junctions, or Gap Junctions (GJ), are structures strongly related to processes of neuronal synchronization. These mechanisms of non-synaptic action, also acclaimed as electrical synapses, have been proposed as responsible for processes of synchronization of the globus pallidus and nigrostriatal pathways, and are directly related to PD.^25^ In the striatum and cortex, the GJ between interneurons consists of a quaternary protein with subunits of Connexin-36 hemichannels (Cx36). These proteins allow ions to pass between adjacent cells and, therefore, can propagate electrical activity quickly and synchronously. In several diseases involving neuronal injury, such as epilepsy or stroke there is an association with connexin remodeling^25^, ^26^ and its activity in the modulation of synchronism.^25^ Therefore, verifying the changes in the expression of these proteins becomes essential for understanding the underlying mechanisms of the synchronizing electrical activity in PD. Therefore, the authors suspected that changes in the expression of connexins and hemichannels, even if not coupled, can interfere with local ionic transport, facilitating or hindering synchronization mechanisms. In order to assess possible changes in the expression of Cx36 in the nigrostriatal region of the 6-OHDA treated rats, an optical densitometry analysis of the immunoreactivity to Cx36 was performed. The slices showed immunoreactive puncta both in the lesioned region and in the contralateral hemisphere when compared to the control. The results of the univariate analysis showed a significant difference between the evaluated areas (ipsi- and contra-lateral to the lesion) and between the DP24 and Control groups, confirming the qualitative analysis (lesional area: 0.154 ± 0.002 fraction of pixels per area; contralateral area: 0.142±0.002 fraction of pixels per area; control: 0.127 ± 0.004 fraction of pixels per area; F(2.26) = 22.45; *p* < 0.0001, ANOVA). These results demonstrate that even the contralateral region suffered a positive modulation in the expression of connexin-36, due to the injection of 6-OHDA, when compared to the same region in the control group, as can be seen in Fig. 5.

## Discussion

The results presented in this work indicate significant changes in mechanisms not directly related to synaptic neurotransmission, but associated with the regulation of ionic levels, increased expression of connexins, and exacerbation of glial reactivity after injury induced by 6-OHDA in the nigrostriatal pathways, suggesting that could be involved in the modulation of nigrostriatal pathways. The hypothesis raised is that changes in the expressions of these non-synaptic mechanisms could unbalance the functions mediated by GABA on nigrostriatal circuits, leading to the initiation and execution of the neurodegenerative process and symptoms of PD.

The high prevalence of synchrony, bursts, and low-frequency oscillations across the cortico-basal-ganglia circuit in the brain of patients with PD and the suspicion of their close relationship with motor symptoms[25] is an important and topical issue. As has already been demonstrated for epilepsy, a neurological pathology also involving synchrony, bursting, and low-frequency oscillations, mechanisms not directly involved with synaptic neurotransmission, usually referred to as non-synaptic mechanisms, have been identified for their predominant effects. Here, the authors used the unilateral intra-striatal 6-OHDA lesion in rats to investigate the induced changes in the expression of the NKCC1 and KCC2 (co-transporters) and Na^+^/*K*^+^-ATPase (Na/K pump), whose actions are directly related to the maintenance of cellular ionic transmembrane gradients. The authors also investigated the glial reactivity after the injury and the changes in the expression of connexin-36, a protein implicated in inter-neuronal. The present study shows prominent glial reactivity and increased expression of connexin-36 in 6-OHDA treated animals. Dopaminergic cell death was observed through the significant reduction in the immunoreactivity to the enzyme Tyrosine Hydroxylase (TH), limited to a region of the nigrostriatal pathway ipsilateral to the lesion. Tyrosine Hydroxylase (TH) is the rate-limiting enzyme for the synthesis of dopamine. The hypothesis that the injection of 6-OHDA causes the death of dopaminergic neurons in the striatum is based on the decrease in the number of TH + neurons.^26^ In the present study, a dose of 24 mg of 6-OHDA deployed to the striatum is consolidated as a classic model to promote experimental PD in rats.^16^^,^^26^ According to,^26^ cell death is the result of cytotoxicity induced by 6-OHDA. In the present study, gliosis, characterized by an increased immunoreactivity to GFAP, accompanies the process of cell death. Studies have shown that gliosis is present in PD and astrogliosis has already been considered a therapeutic target for PD.^27^ Increased immunoreactivity to GFAP, associated with the presence of hyper-reactive astrocytes, found in the present study, is in line with the studies.^16^ Since the present findings are restricted to 15 days after 6-OHDA, the microglial and astrocytic hyperplasia, and hypertrophy are restricted to this moment and may change for a different temporal phase. The increase in GFAP immunoreactivity prior to the robust decrease in TH immunoreactivity was described in the animal model of 6-OHDA, with the presence of glial cells with enlarged cell bodies and reactive cytoplasmic projections throughout the lesioned region and its surroundings. This glial proliferation has been related to the pro-inflammatory processes of PD, since its onset and neurodegenerative progression.^28^ In more advanced cases, glial scarring, or astrogliosis, can form at moderate or even severe levels.^29^ In human tissues, hyperplastic and hypertrophic reactive astrocytes, increased number, and size of astroglial processes, and increased GFAP synthesis have been reported,^28^ similarly as can be observed in Fig. 4, in the slice of animal submitted to the injection of 6-OHDA.

The presence of reactive astrocytes in the ipsilateral and contralateral hemispheres was also observed in other brain regions such as the gray matter, frontal cortex, parietal, piriformis and septum; especially in analyzes performed one week after surgery.^16^^,^^26^ As stated in previous studies, this “distant” reaction seems to reflect the diverse role of the glial population, related to the synthesis of neurotrophins, growth factors, synaptogenesis, and plasticity,^28^ in addition to oxidative stress and accumulation of alpha-synuclein and lipofuscin.^30^ The appearance of cytoplasmic corpuscles, similar to lipofuscin, in cells with small nuclei, reinforces the observation of oxidative stress along the nigrostriatal pathway. As reported.^31^ oxidative stress directly impacts glial functioning, since lipofuscin is commonly found in neurons, but occasionally detected in glial cells, being seen only in processes of oxidative stress and autophagy, leading to the accumulation of non-digestible lipid and protein material within the lumen of the autolysosomes.^31^ In the present study, auto-fluorescent corpuscles like lipofuscin, in addition to being enlarged, are more numerous and distributed, both in the lesional region and in the regions where adjacent glial proliferation was observed, with characteristics like lysosomal granules. These accumulations can be toxic^31^ and be directly related to the role of alpha-synuclein aggregation in PD neurodegeneration.^30^ There is ample evidence that both oxidative stress and neuroinflammation contribute to the neuronal degeneration seen in PD.^32^ It has been observed, in animal models of PD, that brain neuroinflammatory mechanisms lead to a cascade of events that contribute to neurodegeneration.^5^ Severe insults to brain tissue and neurodegenerative characteristics can lead to exacerbated glutamate release and increased intracellular calcium, with consequent activation of signaling cascades for cell death, triggered by excitotoxicity.^33^ After the inflammatory process, reorganization of neuronal circuitry and the glial substrate, within the injured regions, can lead to an imbalance in the inhibitory/excitatory neurotransmission and contribute to **a** lower seizure threshold and increase synchronizing capacity.^34^ In the present study, the presence of lipofuscin, in addition to the high glial reactivity in the 6-OHDA-lesioned region may suggest nigrostriatal synchronizing imbalances.

The striatum, as well as other structures of the nigrostriatal pathways, have been related to important somatic motor processes. Such processes, even under normal physiological conditions, demand synchronization in certain circuits with the basal nuclei and mesencephalic substantia nigra (theta rhythm, beta and gamma oscillations, and sharp-wave ripples).^21^ Therefore, the processes of excitation and inhibition must be strictly regulated through the inhibitory activity of interneurons, in order to prevent this synchronization from becoming excessive or allowing desynchronizing events,^10^ leading to an imbalance in motor control, as seen in PD. Accordingly, inhibitory and excitatory imbalances in the basal ganglia circuitry can be part of a cascade of unwanted events that culminate in the asynchronous activities observed in PD as already described in previous studies.^35^ Fast-spiking interneurons have been shown to be responsible for processes of synchronization of pyramidal cell populations in the cortex,^36^ hippocampus and striatum.^37^ Due to their high interconnectivity through chemical synapses^38^ and GJ,^39^ afferent excitation in these cells can generate Inhibitory Postsynaptic Potentials (IPSP) synchronized in thousands of pyramidal cells, generating the so-called tonic inhibition.^36^ GABAergic synapses are critical in modulating the basal ganglia neural network and controlling synchronization. GABAergic potentials mediated by the GABA-A receptor can become depolarized due to alterations in non-synaptic mechanisms responsible for chloride homeostasis, such as the KCC2 and NKCC1 cotransporters.^11^ An experimental study in humans showed that bumetanide, a selective blocker of the NKCC1 cotransporter, can control the giant GABAergic Currents (CGG) observed in medium spiny neurons in PD patients.^14^ Through approaches in which these currents are abolished, typical motor symptoms of PD are reduced. These studies suggest that drugs that block CGG may provide a new therapeutic option for PD.^14^ These findings also suggest that the expressions of the cation-chloride cotransporters NKCC1 or KCC2 may be altered in PD. The fact that in the present study, after 15 days of the 6-OHDA injection into the striatum, no changes were observed in the expression of the NKCC1 and KCC2 cotransporters may be associated with the time required for inflammatory processes to lead to changes in the expression of these mechanisms. In inflammatory processes caused by pilocarpine injection, alterations in these co-transporters can be observed only during the first days after the lesion, with the expression of the cotransporters returning to the control values in the second week after the insult, which remained until the eighth week.^40^ These observations allow us to speculate that cotransporters may undergo time-dependent variations in their expression during inflammatory processes or after brain damage. Therefore, it would be important to propose investigations to verify changes in these mechanisms in the first days after neurotoxin injection. Several other studies led to the hypothesis that there could be some alteration in the functionality of Na^+^/*K*^+^-ATPase, due to the dopaminergic deficit evidenced in PD. Some studies have shown that Na^+^/*K*^+^-ATPase and KCC2 are functionally linked, acting together to form a metabolon, a protein structure associated with transmembrane ionic transport.^11^ In different conditions of metabolic stress, such as PD, mitochondrial defects and production of oxidative stress occur, which can lead to a reduction in Na^+^/*K*^+^-ATPase activity.^41^ Despite robust evidence, the present study did not find alterations in the expression of Na^+^/*K*^+^-ATPase. As far as the authors know, there are no previously published works that investigated the effects of 6-OHDA injury on mechanisms such as the Na/K pump and co-transporters. Therefore, it was not possible to determine when or if at some point, after the injection of 6-OHDA in Wistar rats, alterations occur in Na^+^/*K*^+^-ATPase and in chloride Cation-Chloride Cotransporters (CCC). However, the hypothesis about the time of alteration of these non-synaptic regulatory mechanisms of ionic homeostasis still deserves attention when considering reported findings^42^ that showed changes in Na^+^/*K*^+^ -ATPase activity only during the chronic period of the pilocarpine injurious insult. Therefore, future studies should evaluate non-synaptic mechanisms at different 6-OHDA-post injury periods. In addition to CCC, GJ represents another mechanism responsible for the interconnectivity and synchronism processes of striatal fast-spiking interneurons, which are critical for motor behavior.^43^ In this work, the authors observed an increase in immunoreactivity to connexin-36 (Cx36) in the ipsilateral regions of the 6-OHDA lesion and contralateral compared to controls (saline). Considering that GJs act as electrotonic neuronal couplers, the increase in Cx36 may constitute an important means of a progression of the synchronous activities of PD.^44^ Astrocyte activation is associated with increased expression of GJ and hemichannels suggesting that glial and neuronal communication through GJ amplifies neurodegenerative processes.^44^ This is in line with the results of the present study, suggesting that previous astrogliosis was responsible for remodeling the Cx36, which, in turn, corroborates the observation of gliosis spreading beyond the area of cell death. The Cx36 establish an electronic coupling between neurons, therefore, they are involved in neuronal synchronization and play an important role in the formation of electrical synapses.^43^ Electrical synapses containing Cx36 have been shown to play an important role in facilitating the synchronization or phase-blocking activity of neuronal networks, underlying a variety of cognitive and motor processes.^45^ Several other studies demonstrated the involvement of connexins in several CNS disorders, including PD, and this appears to cause an increase in synchronism and oscillatory activities.^22^ Electrical synapses have been associated with several physiological aspects of brain functioning, as well as with anomalous population activities.^46^ The authors^46^ emphasize that a fundamental aspect of electrical neurotransmission mediated by Cx36 is its plasticity since the conductance of the neuron-neuron channels formed can be easily regulated. However, the sensitivity of coupling via Cx36 in relation to the intracellular concentration of ions and protons has not been studied in detail. GJ between interneurons in the striatum and cortex are formed by Cx36 and suggests that their coupling, in rat striatum, in 6-OHDA models of PD, may be up to four times increased.^47^ However, this needs to be further elucidated. The author also correlates GJ with synchronism and oscillatory activities of the basal ganglia circuit, contributing to the pathophysiology of PD. These studies have incorporated GJs into a basic computational model based on basal ganglia conductance to examine their potential influence on timing.^47^ Their findings suggest the existence of GJs between GABAergic neurons in the GPe and Gpi that undergo redistribution due to neural damage in PD and exhibit regulated conduction after dopamine depletion. It is noteworthy that the existence of numerous high-conductance GJs in the Gpe may decrease the ability of pallidal neurons to desynchronize cortical inputs, and that cortical inputs affect synchronization when numerous GJs were present. Corroborating the results of the present study, it was reported^25^ an increase in Cx36 in the striatum of patients with PD, evaluated in postmortem tissues. Through the analysis of Cx36 expression in basal ganglia, it was shown that GJ exists between GABAergic neurons of the External Globus Pallidus (Gpe) and Internal Globus Pallidus (Gpi) and is redistributed due to neuronal damage as in PD.^25^ Furthermore, computer simulations showed that Cx36 with increased conductance could contribute to a decrease the ability of pallidal neurons to correctly desynchronize input stimuli. Furthermore, cortical inputs have been shown to impact synchronization when numerous GJ are present.^25^ The intermittent synchronism that is observed in PD may be the result of a propensity of the basal ganglia circuitry to engage in the brief episodes of synchronization necessary for movement control.^47^ The increased expression of Cx36, observed in the present work, suggests that future studies should focus on non-synaptic mechanisms, especially on the neuronal synchronization mediated by GJ coupling and the consequent impact on the core circuits that modulate the basal ganglia functioning.

It is known that, in addition to inducing PD symptoms, 6-OHDA has some limitations as an experimental model. As already reported in the literature, 6-OHDA causes damage to other parts of the brain, it does not provide the production of Lewy body-like inclusions,^48^ unilaterally injured rodents exhibit drug-induced rotational behavior,^49^ motor impairments are also observed, mainly due to impairment of limbs contralateral to the hemisphere in which 6-OHDA is administered.^50^ However, despite these disadvantages, the present work provides valuable information about the possible role of non-synaptic mechanisms in PD.

There is plenty of evidence that abnormal synchronized oscillatory activity involving basal ganglia circuitry is related to motor dysfunction in PD. These observations suggest non-synaptic mechanisms as new potential therapeutic targets. One possibility would be to use the GJ blockers directly along the nigrostriatal pathways, in order to reduce/control aberrant synchronism and, thus, control PD motor symptoms. In addition, protocols for investigating the electrophysiology of the striatum and the substantia nigra using in vitro preparations, in order to verify changes in neuronal synchronism, comparing brain slices of control animals and experimental models of PD, are needed.

## Authors contributions

Mônica C.P. Viegas: Performed experiments and performed the analysis.

Luiz E.C. Santos: Performed experiments; performed the analysis and wrote the original draft preparation.

Mayra C. Aarão: Performed the analysis.

Samyra G. Cecilio: Supervised the experiments.

Joana M. Medrado: Contributed with the immunohistochemistry.

Arthur C. Pires: Contributed with the image processing analysis.

Antônio M. Rodrigues: Contributed with the image processing analysis.

Carla A. Scorza: Contributed with the immunohistochemistry analysis.

Marcelo A. Moret: Contributed with the image processing analysis.

Jose Finsterer: Writing-Reviewing.

Fulvio A. Scorza: Designed and directed the project.

Antônio-Carlos G. Almeida: Conceived and planned the experiments, designed and directed the project and writing-reviewing manuscript.

## Declaration of Competing Interest

The authors declare no conflicts of interest.
